# Supplementary material for: Doping Ferrocene-Based Conjugated Microporous Polymers with 7,7,8,8-Tetracyanoquinodimethane for Efficient Photocatalytic CO2 Reduction
Source: Molecules. 2024 Apr 11;29(8):1738. doi: 10.3390/molecules29081738 (PMC11052251; doi:10.3390/molecules29081738)
Supplement: Supplementary file 1 [file molecules-29-01738-s001.zip › molecules-2953436-supplementary.pdf]

# Supplementary Materials

# Doping Ferrocene-Based Conjugated Microporous Polymers with 7,7,8,8-Tetracyanoquinodimethane for Efficient Photocatalytic $CO_2$ Reduction

Shenglin Wang [1,†], Qianqian Yan [1,†], Hui Hu [1,*], Xiaofang Su [1], Huanjun Xu [2], Jianyi Wang [1] and Yanan Gao [1,*]

1 Key Laboratory of Ministry of Education for Advanced Materials in Tropical Island Resources, Hainan University, No 58, Renmin Avenue, Haikou 570228, China; wangshenglin@hainanu.edu.cn (S.W.); yanqianqian@hainanu.edu.cn (Q.Y.); sxf@hainanu.edu.cn (X.S.); energywang@hainanu.edu.cn (J.W.)

2 School of Science, Qiongtai Normal University, Haikou 571127, China; xuhuanjun86@iccas.ac.cn

* Correspondence: hhu@hainanu.edu.cn (H.H.); ygao@hainanu.edu.cn (Y.G.)

† These authors contributed equally to this work.

## Methods

### Characterization.

Matrix-assisted laser desorption ionization time-of-flight mass (MALDI-TOF MS) spectra were recorded on an Ultraflextreme spectrometer with the substrate was 2,5 dihydroxybenzoic acid. Solid-state diffuse reflectance spectra (UV-Vis DRS) were recorded on a JASCO model V-770 spectrophotometer by measuring the reflectance of powders in the solid state. Fourier-transform infrared (FT IR) spectra were detected on a JASCO model FT/IR-6800 infrared spectrophotometer with a scan number of 16, and the background was subtracted. $N_2$ adsorption analyses were performed by using Quantachrome Autosorb-IQ2 to analyze the specific surface area and pore size distributions. Before measurement, powder samples were degassed under a dynamic vacuum at 120 °C for 15 h. Brunauer-Emmett-Teller (BET) surface areas were calculated from the linear region of the $N_2$ isotherm at 77 K within the pressure range $P/P_0$ of 0.003–0.05 using micropore BET assistant on the ASiQwin software. Pore size distributions were determined using using the quenched solid density functional theory (QSDFT) method. Thermogravimetric analysis (TGA) was recorded on a Mettler TG-DSC 3+ under $N_2$ at a heating rate of 10 °C min$^{-1}$ from ambient temperature to 800 °C. High-resolution transmission electron microscope (HR-TEM) analysis was recorded on FEI Tecnai G2 F30 electron microscope. X-ray photoelectron spectroscopy (XPS) measurements were performed on a Thermo escalab 250Xix spectrometer with Al K$\alpha$ X-rays as the excitation source and the C1s peak at 284.6 eV as the reference line. The photoluminescence spectroscopy (PL) was collected on a JASCO FP-8600 spectrofluorimeter with 450 nm excitation. Fluorescence lifetime was collected on a Quantaurus-Tau C16361 spectrofluorimeter with 405 nm excitation. MALDI-TOF-MS were performed using an UltrafleXtreme MALDI MS instrument.

### Synthesis of model compound.

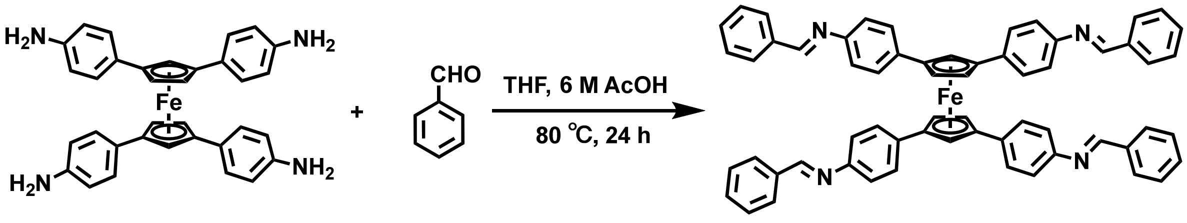


**Scheme S1.** Synthetic route of the model compound.

TAPFc (50.1 mg, 0.09 mmol), benzaldehyde (38.2 mg, 0.36 mmol), THF (5 mL), and 6 M AcOH (0.5 mL) were added to a 25 mL reaction tube, and the mixture was heated and stirred at 120 °C for 24 h under nitrogen and then cooled to room temperature. The solvent was removed under reduced pressure, and the solid was ultrasonically washed with ethyl acetate, filtered, and dried under vacuum at room temperature for 2 h to obtain an orange-red model compound in 60% yield. NMR spectrum of the model compound could not be recorded due to its limited solubility in common deuterated solvents. The MALDI-TOF-MS spectra of the model compound was shown in Figure S1.

**Model compounds react with TCNQ.**

After adding the model compound (10.0 mg) to a 10 mL reaction tube, TCNQ (1:2 or 1:4 molecular ratio) in different molar ratios was added, and the mixture was heated for 24 h at 60 °C in dichloromethane (5 mL).

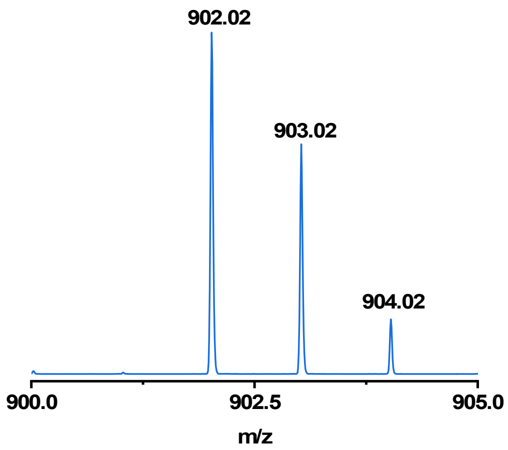


**Figure S1.** MALDI-TOF-MS spectrum of the model compound.

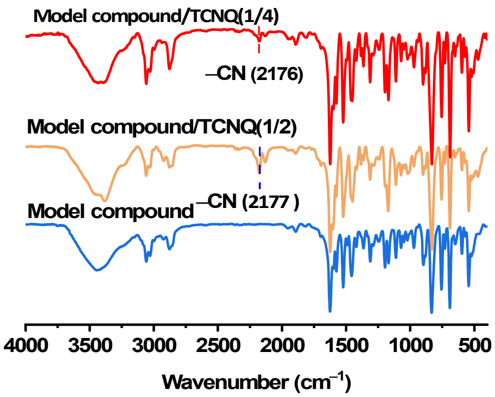


**Figure S2.** FT-IR spectra of the model compound and its complexes with TCNQ in different molar ratios.

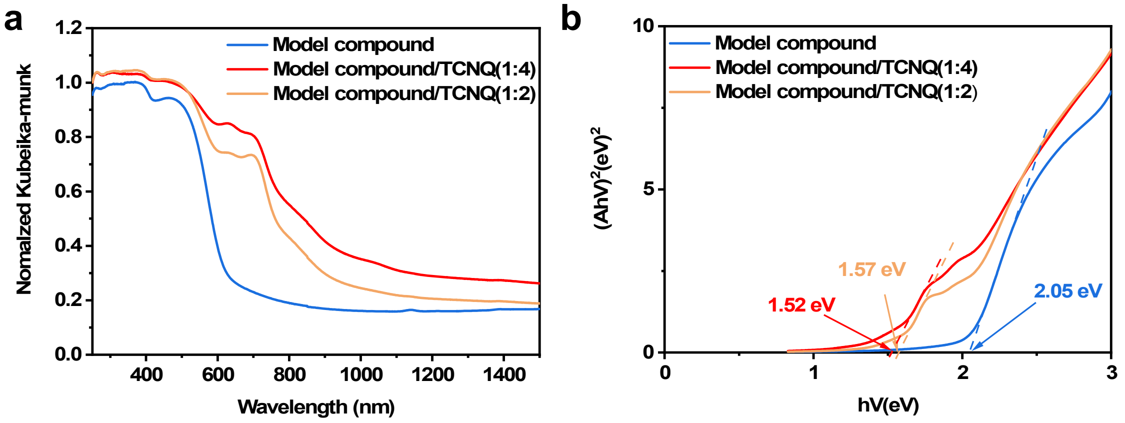


**Figure S3.** (a) UV-Vis DRS and (b) Tauc plots of the model compound and its complexes with TCNQ in different molar ratios.

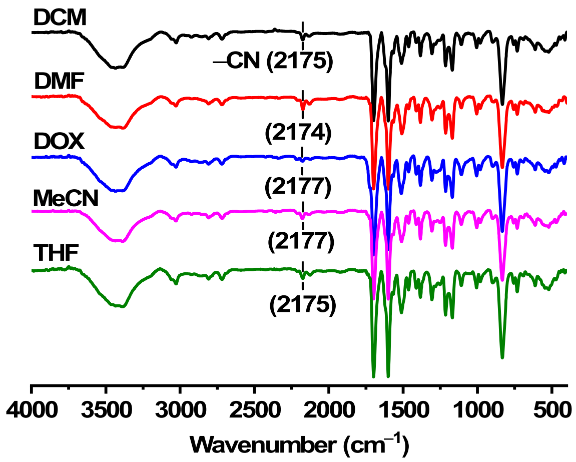


**Figure S4.** FT-IR spectra of CMPs doped with TCNQ (1:4, mass ratio) in different solvents.

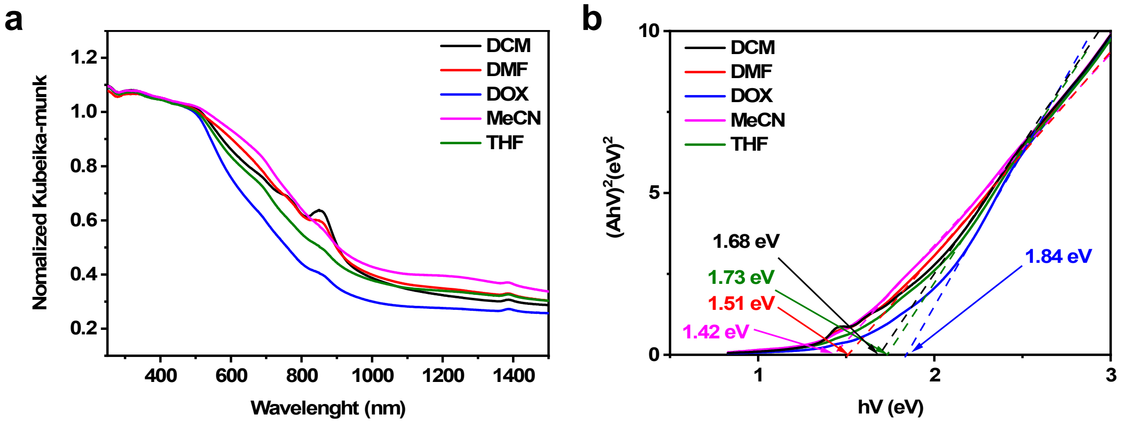


**Figure S5.** (a) UV-Vis DRS and (b) Tauc plots of CMPs doped with TCNQ in different solvents.

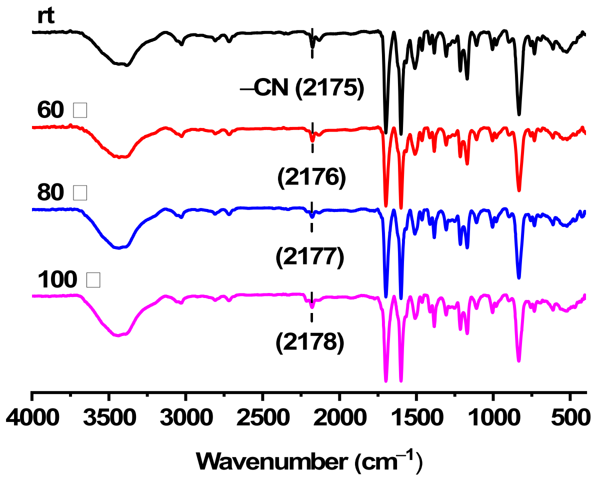


**Figure S6.** FT-IR spectra of TCNQ doped on the CMP at different temperatures.

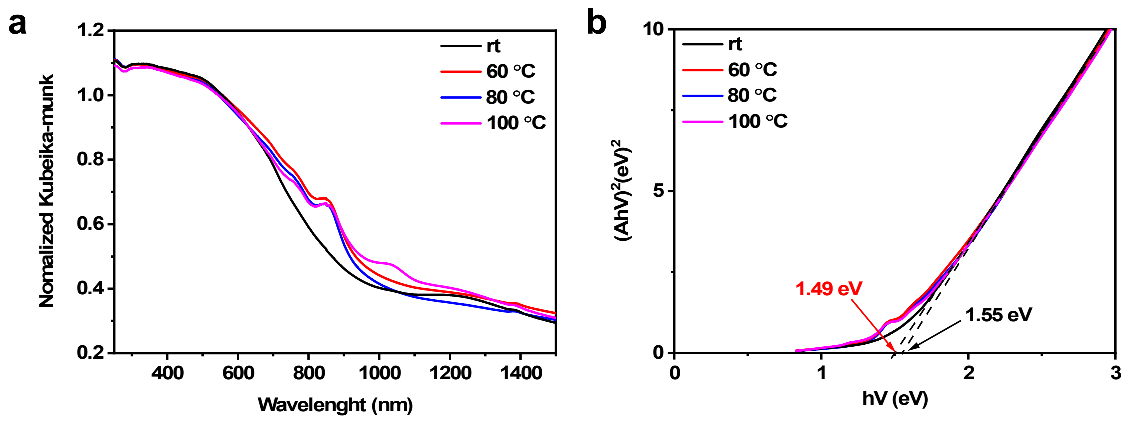


**Figure S7.** (a) UV-Vis DRS and (b) Tauc plots of TCNQ doped on the CMP at different temperatures.

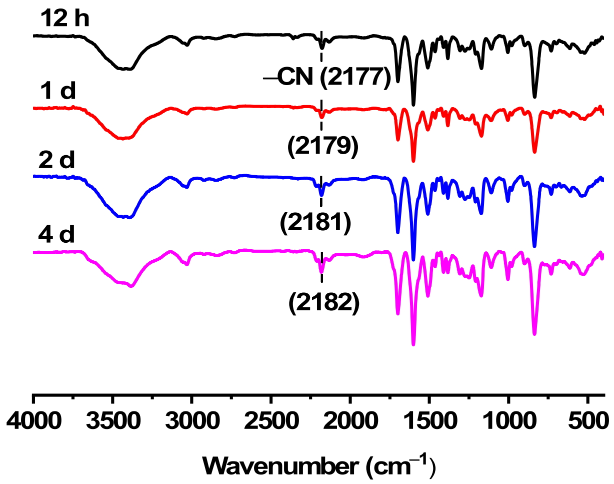


**Figure S8.** FT-IR spectra of TCNQ doped on the CMP for different duration times.

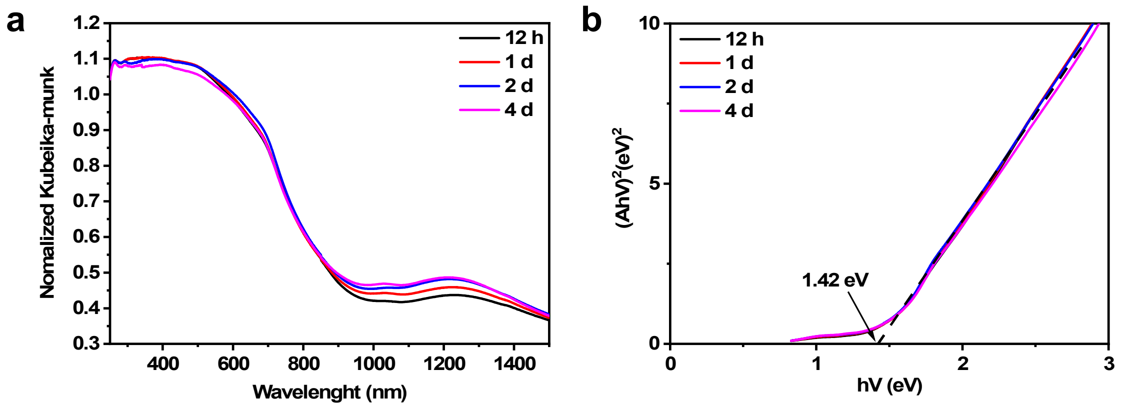


**Figure S9.** (a) UV-Vis DRS and (b) Tauc plots of TCNQ doped on the CMP for different duration times.

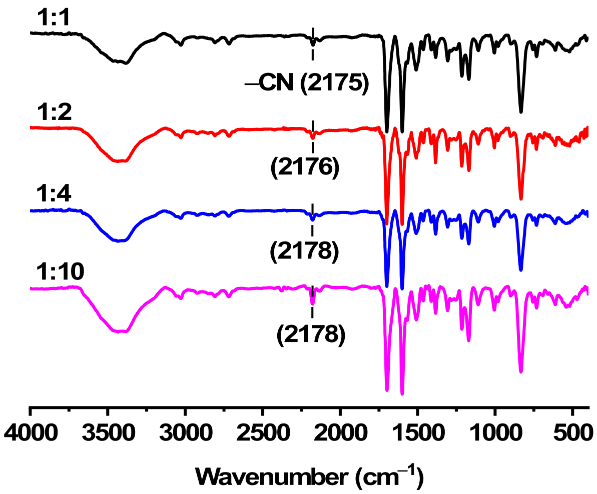


**Figure S10.** FT-IR spectra of TCNQ doped on the CMP at different TCNQ dosages (mass raito).

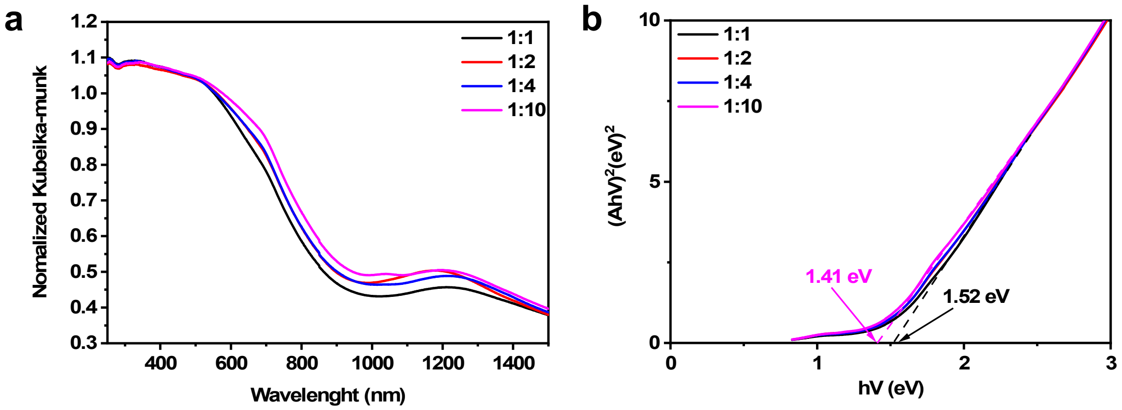


**Figure S11.** (a) UV-Vis DRS and (b) Tauc plots of TCNQ doped on the CMP at different TCNQ dosages (mass raito).

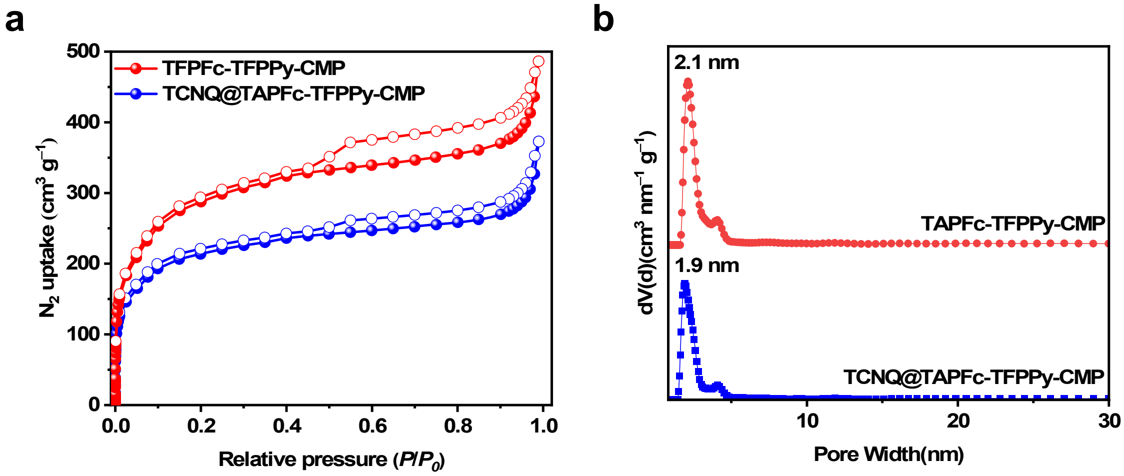


**Figure S12.** (a) $N_2$ sorption isotherms at 77 K and (d) pore size distribution curve of TAPFc-TFPPy-CMP (red) and TCNQ@TAPFc-TFPPy-CMP (blue).

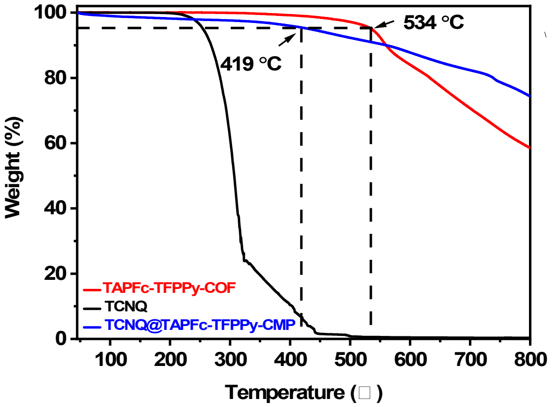


**Figure S13.** TGA curves of TAPFc-TFPPy-CMP (red), TCNQ@TAPFc-TFPPy-CMP (blue) and TCNQ (black).

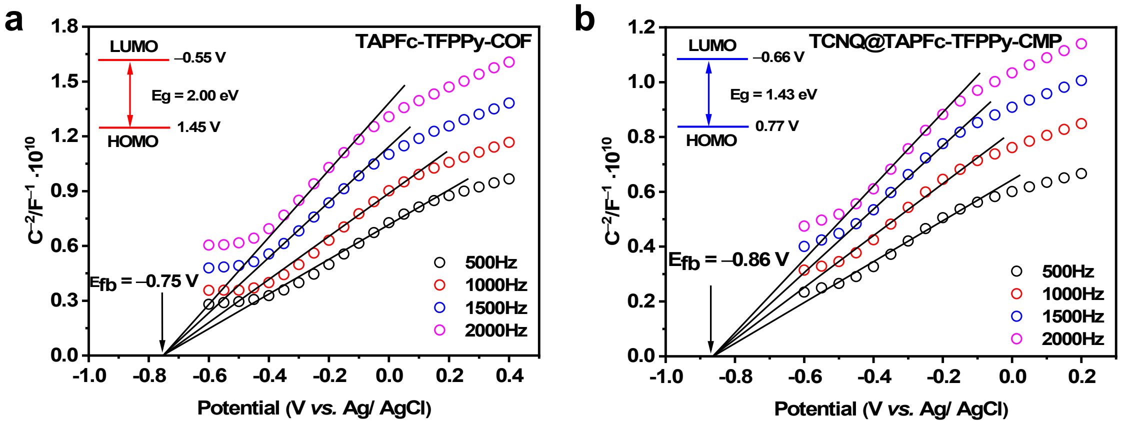


**Figure S14.** Mott-Schottky curves of TAPFc-TFPPy-CMP (a) and TCNQ@TAPFc-TFPPy-CMP (b).

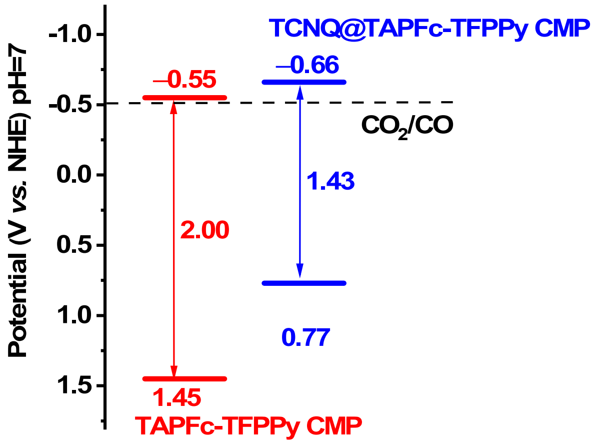


**Figure S15.** Energy band structure of TAPFc-TFPPy-CMP (red) and TCNQ@TAPFc-TFPPy-CMP (blue).
